# Micro- and macrocirculatory effects of norepinephrine on anaesthesia-induced hypotension: a prospective preliminary study

**DOI:** 10.1186/s12871-023-02342-3

**Published:** 2023-11-16

**Authors:** Manuel Kindermans, Jona Joachim, Elsa Manquat, Charlotte Levé, Alex Hong, Joachim Mateo, Alexandre Mebazaa, Etienne Gayat, Daniel De Backer, Fabrice Vallée

**Affiliations:** 1 Department of Anaesthesiology and Intensive Care, Lariboisière - Saint Louis Hospitals, 02 rue Ambroise Paré, 75010 Paris, France; 2 Inserm, UMRS-942, Paris Diderot University, 02, rue Ambroise Paré, 75010 Paris, France; 3 CHIREC, Brussels, Belgium; 4 Intensive Care Department, CHIREC Hospitals, Brussels, Belgium; 5 Inria Saclay Ile-de-France, Palaiseau, France; 6 LMS, École Polytechnique, CNRS, Paris, France

**Keywords:** Norepinephrine, Bolus, Continuous infusion, Microcirculation, Microcirculation, Microflow index, Total vessel density

## Abstract

**Background:**

Intraoperative arterial hypotension (IOH) leads to increased postoperative morbidity. Norepinephrine is often use to treat IOH. The question regarding the mode of administration in either a bolus or continuous infusion remains unanswered. The aim of the present study was to describe and compare the effects on macrocirculation and microcirculation of a bolus and a continuous infusion of norepinephrine to treat IOH.

**Methods:**

We conducted a prospective observational study with adult patients who underwent neurosurgery. Patients with invasive arterial blood pressure and cardiac output (CO) monitoring were screened for inclusion. All patients underwent microcirculation monitoring by video-capillaroscopy, laser doppler, near-infrared spectroscopy technology, and tissular CO_2_. In case of IOH, the patient could receive either a bolus of 10 µg or a continuous infusion of 200 µg/h of norepinephrine. Time analysis for comparison between bolus and continuous infusion were at peak of MAP. The primary outcome was MFI by videocapillaroscopy.

**Results:**

Thirty-five patients were included, with 41 boluses and 33 continuous infusion.

Bolus and continuous infusion induced an maximal increase in mean arterial pressure of +30[20-45] and +23[12-34] %, respectively (*P*=0,07). For macrocirculatory parameters, continuous infusion was associated with a smaller decrease in CO and stroke volume (p<0.05).

For microcirculatory parameters, microvascular flow index (-0,1 vs. + 0,3, *p*=0,03), perfusion index (-12 vs. +12%, *p*=0,008), total vessel density (-0,2 vs. +2,3 mm2/mm2, *p*=0,002), showed significant opposite variations with bolus and continuous infusion, respectively.

**Conclusions:**

These results on macro and microcirculation enlighten the potential benefits of a continuous infusion of norepinephrine rather than a bolus to treat anaesthesia-induced hypotension.

**Trial registration:**

(NOR-PHARM: 1-17-42 Clinical Trials: NCT03454204), 05/03/2018

## Key points

At the macrocirculatory level continuous infusion resulted in a smaller decrease in CO and SV and a smaller increase in afterload than bolus administration.

At the microcirculatory level continuous infusion enhanced blood flow and vessel density while these variables were impaired with a bolus.

These results on macro and microcirculation enlighten the potential benefits of a continuous infusion of norepinephrine rather than a bolus to treat anaesthesia-induced hypotension.

## Introduction

Intraoperative arterial hypotension (IOH) leads to increased postoperative morbidity [1–3]. During the induction and maintenance of anaesthesia, IOH is often related to a decrease in vascular tone induced by hypnotic and morphinomimetic drugs [4]. This condition, vasoplegia, is also associated with impaired microcirculatory perfusion [4, 5].

Vasopressor agents, especially norepinephrine, are commonly administered to correct IOH [6]. In the operating room, norepinephrine is sometimes administered in a bolus even though continuous infusion may be preferred [7–10]. In fact, even when effective in rapidly restoring adequate arterial blood pressure (ABP), the bolus mode of administration is also associated with a decrease in heart rate (HR) and cardiac output (CO). This comes with increased arterial stiffness and left ventricular afterload [7, 9, 11]. As CO is an important determinant of tissue perfusion [12], one could imagine that those effects could be transposed at the microcirculatory level and therefore lead to impaired tissue perfusion and oxygenation [13].

These constatations could have clinical implications: recently, continuous infusion of norepinephrine has been associated with better control of peroperative ABP and better renal postoperative outcomes than boluses [8].

Microcirculation monitoring has been intensely developed since it became a topic of interest more than 20 years ago. Indeed, microcirculation has proven to be strongly associated with organ dysfunction and outcome in various shock models [12, 14, 15]. Even once macrohemodynamics are restored, microcirculation can remain altered, and this condition is also associated with a worse outcome [16]. These results have only been published on populations of ICU patients. However, links between microcirculation and postoperative outcomes have been established [17].

Devices now available for microcirculatory monitoring present each advantages and disadvantages. Nowadays laser doppler, near-infrared spectroscopy technology (NIRS), tissular CO_2_ and videocapillaroscopy are mostly used [18]. Again, initially more frequent in ICUs, their use in the operating room becomes increasingly common [13, 19].

Despite these elements, the effects of norepinephrine in boluses and continuous infusion remains unknown in patients undergoing anaesthesia. To our knowledge, the combined description of macro- and microcirculatory effects of norepinephrine has only been studied in shocked patients, showing various and heterogeneous results [20–22].

The aim of the present study was then to describe and compare the effects of a bolus and a continuous infusion of norepinephrine on macrocirculation and microcirculation to treat anaesthesia-induced hypotension.

The hypothesis was that continuous infusion could result in an improved global cardiovascular adaptation than a bolus on both macro- and microcirculatory parameters.

## Material and methods

### Ethics

This study was approved by the Ethics Committee of Agence Régionale de Santé Occitanie (FRANCE, president D. Benayoun) (NOR-PHARM: 1-17-42 Clinical Trials: NCT03454204) (05/03/2018). This prospective observational study was conducted in the Department of Anaesthesiology and Critical Care of the University Hospital of Lariboisière, Paris, France. All methods were carried out in accordance with relevant guidelines and regulations. Written informed consent was obtained from all subjects, and/or their legal guardian(s), participating in the trial.

### Patients

The inclusion criteria were as follows: adult patients (> 18 and older) undergoing major surgery (mostly neurosurgical interventions). Only patients for whom preoperative anaesthesia consultation indicated continuous ABP and CO monitoring were screened for inclusion.

The exclusion criteria were as follows: patients aged under 18 years old, pregnancy, haemodynamic instability caused by acute bleeding, critical haemodynamic instability, sitting position and preoperative doses of norepinephrine > 500 µg/hour.

### Patients’ characteristics and management

All cardiovascular, respiratory, and neurologic comorbidities, ASA (American Society of Anaesthesiologists) scores, usual medications, clinical abnormalities, and results of preoperative diagnostic tests were recorded on the anaesthesiology report. Patients were treated in accordance with local care protocols.

All general anaesthesia were conducted under intravenous administration of propofol and remifentanil guided by AIVOC mode (with Schneider model for propofol and Minto model for remifentanil). The bispectral index guided the deepness of anaesthesia with objectives between 40 and 60. Patients’ lungs were mechanically ventilated in volume control mode (tidal volume: 6 ml/kg) with an O_2_/air mixture (FiO2 0.4) and a positive end expiratory pressure of 5 cmH_2_O. The respiratory rate was adjusted to keep end-tidal carbon dioxide partial pressure (EtCO_2_) between 35 and 40 mmHg.

Standard monitoring included HR, pulsed oximetry (SpO2), perfusion index (PI) [23], invasive ABP, temperature, and ventilatory settings, including end tidal CO2 (EtCO2). CO and SV monitoring was performed via oesophageal Doppler (Deltex Medical Limited, Chichester, UK).

All variables, including CO and stroke volume (SV) were recorded continuously and extracted via the Xtrend Logiciel from Philips® (Amsterdam, Netherlands) scopes.

Cardiac afterload was monitored via the Global After Load Angle (GALA). Its value was visualized on the transoesophageal Doppler screen and calculated from the velocity pressure loops (VP loops) as previously described [24].

### Microcirculation monitoring

Microcirculation monitoring was performed simultaneously with 5 different devices. All the devices and the main scope monitor were connected and synchronized, allowing to find retrospectively all the desired events as the peak of MAP.

- Videocapillaroscopy using incident dark field imaging device (Braedius®, Netherlands) : intraoperative sublingual video allowed direct visualization of sublingual blood flow in microvascular networks. Images acquisition and analysis were performed according to international guidelines [25]. Care was provided to avoid pressure artefacts and saliva was removed as much as possible before acquisition.

If a bolus was administered, all the sequences were recorded in the same zone one after the other. First sequence was considered as the basal state of patient’s microcirculation. When the peak of MAP was reached, the sequence corresponding was kept apart to be analyzed after. This is in line with the new guidelines [25].

In case a continuous infusion was administered, 5 sequences were recorded in 5 different zones at basal state and 5 supplementary ones were recorded when the arterial blood pressure was stabilized at the next level (plateau). Sequences duration was between 8 and 10 seconds.

Two trained operators (MK, FV) recorded the clips.

Analysis was made by four trained investigators (MK, FV, JM, JJ), independently, and blinded from other’s investigator’s results. They were assisted by the last version of the software CytoCamTools Study Manager provided by Braedius® only concerning the total vessel density score. Vessels were added or removed manually if the detection proposed by the software was not satisfactory.

Clips were not randomized before analysis, but analysis was made blinded from the identity of the patient, the intervention (bolus or continuous infusion), and the level of arterial blood pressure at the time of the recording.

Four microcirculatory parameters have been analyzed:

1. The microvascular flow index (MFI), which is a qualitative evaluation of the microvascular flow. The image is divided into four quadrants, and the predominant type of flow in very small vessels is assessed in each quadrant using an ordinal score (0 = no flow, 1 = intermittent flow, 2 = sluggish flow, 3 = normal flow). The overall score, called microvascular flow index, is the sum of each quadrant score divided by the number of quadrants.
2. The total vessel density (TVD)
3. The perfused vessel density (PVD), which is calculated using the formula: PVD = PPVxTVD.
4. The Heterogeneity Index (HI), calculated as the difference between extreme values of MFI between the five recordings divided by its mean value for the continuous infusion recordings; and as the difference between the best and the worst flow divided on the average flow for bolus recordings.
  Laser Doppler (Transonic System®, Ithaca, USA) : This technique is based on the reflection of a beam of laser light that undergoes a change in wavelength (Doppler shift) when moving blood cells are encountered. The magnitude and frequency distribution of these changes are related to the number and velocity of red cells. The quantitative value derived from these measurements (red blood cell flow) is expressed in arbitrary perfusion units (Tissular Perfusion Unit, TPU).
  Near-Infrared Spectroscopy technology (NIRS), providing tissular saturation in O2 (StO2) (InSpectra®, Hutchinson Technology, Minnesota, USA) : two sensors placed on the patients’ thenar eminence emit near-infrared light in four specific wavelengths (690, 780, 805 and 850 nm), which is then partially absorbed and partially reflected to the sensors, based on the ratio of oxygenated to deoxygenated hemoglobin in the tissue. NIRS also measures THI (tissular hemoglobin index), an index of microcirculatory volume of hemoglobin, with values ranging from 0 to 100.
  Tissular CO2 measure (TOSCA 500, Radiometer®,Copenhague, Danemark) : Transcutaneous measurement of PCO2 is based on the phenomenon of CO2 gas diffusing very easily throughout the body tissue and skin, which can thus be detected by a sensor on the skin surface. tPCO2 reflects tissue perfusion by an evaluation of the difference between tPCO2 and artPCO2, so-called « CO2gap » [26].
  Perfusion Index (PI) by photoplethysmography (Two devices : Phillips® and Masimo®)

Examination of microcirculation with an incident dark field imaging, laser Doppler, NIRS technology and transcutaneous CO2 captor is completely noninvasive and respects the noninterventional study design.

Laser Doppler and tissular CO2 probes were each placed on one ear as previously described in other publications [23]. The NIRS probe was placed on the thenar eminence [27], contralateral to the arterial catheter. Photoplethysmography by Philips® was placed on one index contralateral to the arterial catheter.

### Protocolized management of IOH

The mean arterial blood pressure (MAP) reference was the average between MAP at anaesthesia consultation and MAP the day before surgery. IOH episode was defined as a decrease in MAP under 65 mmHg or below 20% of the MAP reference in the absence of clear hypovolemia. Hypovolemia was defined in our institution by the conjunction of a significant decrease in CO compared to the CO target value obtained after CO optimization before surgical incision, a DeltaPP >15% and/or an acute bleeding episode.

When a patient presented an IOH episode, the anaesthesiologist in charge could:

- Either administered a bolus of 2 mL (10 µg) of norepinephrine diluted at 5 µg/mL.
- Or either introduce a continuous infusion of the same treatment at speed 40 mL/hour (i.e., 200 µg/hour or 16 µg/5 min). If a continuous infusion was already started, an elevation of 40 ml/hour more was added.

The protocol was not randomized. As explained, the decision of a bolus or a continuous infusion was not made by the investigators of the study, but by the anaesthesiologist in charge of all the intervention. Often but not systematically, continuous infusion is given if the arterial pressure continues to be low despite some one or two first boluses. Patients could receive two boluses but it had to be with an interval of more than 10 minutes. Patients receiving a bolus directly followed by a continuous infusion were not included.

### Collection of macro- and microcirculatory parameters over time:

For the bolus, the analysis times were T0, T15, T30, T45, T60 seconds and at peak of MAP. This choice derives from a study we are conducting (nonpublished data) which shows that the peak in MAP is reached at approximately 50 seconds. For videocapillaroscopy sequence analysis, the times were T0 and at the peak of ABP increase.

For the continuous infusion, the analysis times were T0, T180, T300 seconds and at peak of MAP. For videocapillaroscopy, times were T0 and at the ABP plateau (plateau being defined by less than 5% variations in PAM for more than 20 seconds).

Values of each parameter at peak of effect and time to reach this peak were also recorded.

#### Outcomes

The primary outcome MFI measured by videocapillaroscopy. Secondary outcomes were CO, MAP, SV, TVD, PPV, HI, PI, TPU, StO2 and tPCO2.

### Statistics

Statistical analysis was performed with GraphPad Prism® software (La Jolla, California, USA). The significance level was α= 0,05. Data with a normal distribution are presented as the mean ± standard deviation, and those with a nonnormal distribution are presented as the median ± interquartile range. The normality of the population distribution was determined using a Shapiro test. Variations in the parameters over time were always compared with the baseline value using a Wilcoxon test and expressed as a percentage. To compare the values obtained with a bolus and with a continuous infusion at each time point including the peak of effect (peak of MAP), we performed a Mann–Whitney test. As the number of boluses and continuous infusion varied from one patient to another, we used weighted methods to compare those average values, with the weights being the number of measures per subject.

Inter- and intraindividual variability for videocapillaroscopy analysis was assessed by calculating the kappa coefficient of concordance.

## Results

### Patients

Forty-one patients were selected between January 2019 and March 2020. Six were excluded: five due to lack of recording and one due to preoperative doses of norepinephrine > 500 µg/h. Thirty-five patients undergoing neurosurgery (32 patients) or neuroradiology (3 patients) procedures were prospectively included: 27/35 (77%) patients underwent intracranial surgery, and 5/35 (14%) underwent spinal surgery. We had for intracranial surgery : 4 aneuvrism clipping, 9 meningioma removal, 8 single or multiple metastasis removal, 4 high grade tumor removal and 2 schwanomma removal. Main operation duration was 5h30. Spinal surgery was represented bay arthrodesis (4/5) of at least three stages. Patient characteristics are reported in Table 1.

**Table 1:** Patient characteristics

Characteristics	Total Patients *n*=35
Female Gender, no (%)	25 (71)
Age (years)	58 (52-66)
Weights (kg)	70 (60-80)
Size (cm)	165 (162-170)
BMI (kg/m2)	25,7 (22-28)
ASA, n (%)	
I	2 (6)
II	26 (74)
III	7 (20)
Cardiovascular Risk Factors (%)	
High Blood Pressure	11 (31)
Active Tabagism	4 (11)
Diabetes Mellitus	1 (2)
Dyslipidaemia	5 (15)
Obesity	3 (9)
Chronic Kidney Failure	1 (3)
Antecedents (%) Treatments (%)	
Solid Cancer	8 (23)
ACE-I /angiotensin blockade	5 (15)
Calcium Channel Blocker	3 (9)
Beta blocker	3 (9)
Diuretics	2 (6)
Biology	
Preoperative Haemoglobin (g/dL)	13,4 (12,4-14,6)
Platelets (g/L)	266 (176-256)
Creatinine (µg/L)	67 (58-80)

**Fig. 1:**
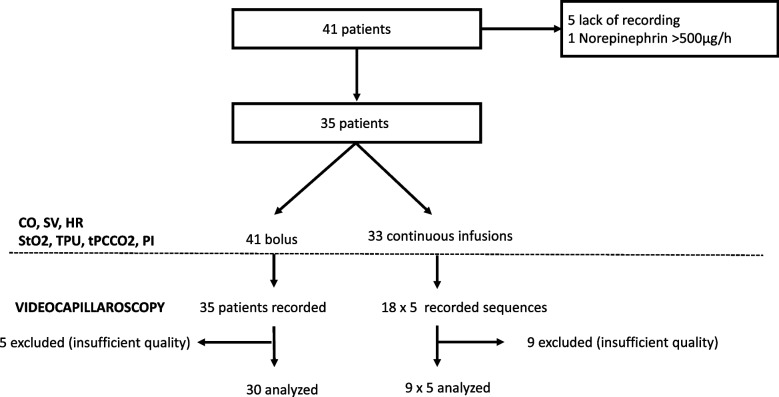
Flow chart

In those patients, we analysed 41 bolus and 33 continuous infusion sequences with complete recording of macrocirculation and microcirculatory devices. Every patient received at least one bolus, 6 patients received two boluses and no patient received more than two boluses. For videocapillaroscopy, 30 bolus sequences were analysed in 30 patients (5 recorded sequences presented insufficient quality), and only 9 sequences of continuous infusion were analysed (over 18 recorded), for the same reason (Fig. 1).

There were no differences between haemodynamic baseline values when comparing bolus and continuous infusion populations. This was consistent for macro and microcirculatory parameters.

### Time to reach maximal effect

Concerning microcirculatory parameters, the maximal effect on MAP occurred 52 [47-55] seconds after the bolus. The time to reach the MAP plateau was 260 [160-303] seconds after continuous infusion started.

### Macrocirculatory parameters

Before bolus and continuous infusion, patients presented a median MAP of 67 (60-74) mmHg, a CO of 4,8 [2, 3, 3-6] L/min and an SV of 74 [49-86] mL, for an HR of 70 [61-77] pulse/min. Variations of pulsed pressure (ΔPP) was 10 [5-12] %. Macrocirculatory parameters before bolus and continuous infusion were not different.

*Effects of a bolus of norepinephrine*: Norepinephrine boluses were associated with a 30 [20-45] % increase in MAP (*p*<0,0001) and a decrease in HR and SV of 6 [1-11] % (*p*<0,0001) and 12 [4-16] % (*p*<0,0001), respectively, leading to a decrease in CO of 15 [16-29] % (*p*<0,0001). GALA increased by +12 [6-15]° (*p*=0,008) in median (Table 2).

**Table 2:** Bolus parameters evolution over time

	**Baseline**	**T15’**	**T30’**	**T45’**	**T60’**
**MACROCIRCULATION**					
MAP (mmHg)	68 (60-74)	68 (61-77)	72 (62-88)*	79 (74-91)*	89 (78-96)*
HR (/min)	70 (61-77)	70 (62-77)	70 (60-72)	66 (60-72)*	64 (58-71)*
CO (L/min)	4,8 (3,2-6)	4,8 (3,1-5,7)	4,6 (2,8-5,7)*	3,9 (2,6-5,4)*	3,9 (2,9-4,8)*
SV (mL)	74 (49-86)	70 (46-82)*	70 (42-75)*	62 (42-75)*	58 (43-71)*
GALA (°)	40 (26-47)				46 (35-64)
**MICROCIRCULATION**					
**LASER DOPPLER**					
TPU	6,3 (3,7-11,7)	7,3 (3,9-11,6)	6,9 (4,4-12,2)*	6,9 (4,2-10,6)	6 (3,7-11,5)
**PHOTOPLETHYSMOGRPAHY**					
PI (finger) (%)	5,7 (3-8,7)	5,9 (3,1-8,2)	5,7 (2,8-8,3)*	5,3 (2,5-7,8)*	5,1 (2-7,8)*
PI (ear) (%)	1,4 (0,6-1,9)	1,4 (0,6-1,9)	1,4 (0,6-1,9)	1,1 (0,6-1,8)	1,2 (0,7-1,9)
**NIRS**					
StO2 (%)	87 (82-89)	87 (83-88)	87 (83-88)	87 (83-88)	87 (82-88)
THI (%)	12,2 (10,4-13,7)	12,3 (10,5-13,6)	12,4 (10,4-13,6)	12,5 (10,4-13,6)	12,5 (10,3-13,7)
**TISSULAR CO2 (mmHg)**	40 (34-43)	39 (34-43)	40 (34-43)	40 (34-43)	40 (34-43)
**VIDEOCAPILLAROSCOPY**					
MFI	2,16 (1,72-2,64)				2,06 (1,66-2,4)
PPV (%)	65,3 (52,7-74,3)				62,5 (55,6-69,4)
TVD (mm2/mm2)	14,1 (11,9-16,1)				13,9 (11,7-15,4)
PVD (mm2/mm2)	8,8 (6,9-10,1)				8,6 (6,8-10,1)
HI	0,36 (0-0,66)				0,4 (0-0,57)

Data are expressed in median (IQR). *MAP* Mean arterial pressure, *HR* Heart rate, *CO* Cardiac output, *SV* Stroke volume, *GALA* Global afterload angle, *TPU* Tissue perfusion unit, *PI* Perfusion index, *MFI* Microvascular flow index, *PPV* Percentage of perfused vessels, *StO2* Tissular saturation in O2, *THI* Tissular hemoglobin index, *TVD* Total vessel density, *PVD* Perfused vessel density, *HI* Heterogeneity Index. (*) = *p* < 0,05

*Effects of a continuous infusion*: Norepinephrine continuous infusions were associated with a median increase of 23[12-34]% in MAP (*p*<0,0001). This was coupled with a drop in HR of -10[-14; -6]% (*p*<0,001) but not in SV, in which variations remained nonsignificant +2[-7 ;+4]%). However, a significant decrease in CO was observed, of 7 [0-14] % (*p*<0,0001). GALA increased by +7 [3-12]° (*p*=0,007) (Table 3)

**Table 3:** Continuous infusion parameters evolution over time

	Baseline	T15’	T30’	T45’	T60’	T180’	T300’
**MACROCIRCULATION**							
MAP (mmHg)	66 (60-74)	69 (58-78)	68 (58-77)	69 (58-78)	70 (59-80)	79 (76-98)*	83 (77-98)*
HR (/min)	61 (55-72)	61 (54-73)	61 (55-73)	60 (55-72)	59 (54-68)	58 (55-64)*	57 (55-61)*
CO (L/min)	4,2 (2,9-6)	4,2 (3-5,8)	4,1 (2,9-5,7)	4,1 (3,1-5)	4,1 (3-4,5)	3,8(3-5,8)*	3,9 (3-5,6)*
SV (mL)	64 (46-81)	64 (46-80)	64 (45-78)	65 (46-82)	64 (43-68)	62 (45-74)	61 (44-76)
GALA (°)	40 (35-69)						43 (37-59)
**MICROCIRCULATION**							
**LASER DOPPLER**							
TPU	7,1 (1,8-13,4)	7,2 (1,9-13,4)	7,2 (1,9-13,4)	7,0 (1,9-14,3)	7 (2,1-14,7)	7,1 (4,4-14)	7,1 (4,5-12,6)
**PHOTOPLETHYSMOGRAPHY**							
PI (finger) (%)	5,5 (3,2-8,3)	5,3 (2,1-7,8)	5,2 (2,1-7,8)	5,1 (2-8,1)	5 (2,2-8,3)	5,9 (3-8,3)	6,4 (3,3-8)
PI (ear) (%)	1,4 (0,6-1,9)	1,4 (0,6-1,9)	1,4 (0,6-1,9)	1,4 (0,6-1,8)	1,4 (0,7-1,9)	1,8 (0,8-2,1)	1,8 (0,8-2,1)
**NIRS**							
StO2 (%)	87 (82-90)	88 (83-90)	88 (82-89)	88 (82-89)	88 (84-89)	85 (82-89)	85 (82-89) *
THI (%)	12,6 (10,5-14,1)	12,4 (10,6-14,1)	12,4 (10,6-14)	12,5 (10,5-14)	12,4 (10,4-13,8)	12,1 (9,5-13,3)	12,5 (9,9-13,8)
**TISSULAR CO2 (mmHg)**	40 (34-43)	39 (34-43)	40 (34-43)	40 (34-43)	40 (34-43)	39 (33-43)	39 (33-43)
**VIDEOCAPILLAROSCOPY**							
MFI	1,8 (1,7-2)						2,1 (1,8-2,4)
PPV (%)	71,6 (61,2-76,7)						69,4 (60,1-74,6)
TVD (mm2/mm2)	15,1 (14,1-15,5)						17,4 (15,6-18,7) *
PVD (mm2/mm2)	10,1 (9-11,5)						11,8* (10,7-13,1)
HI	0,66 (0,4-1)						0,44 (0,36-0,8)

Data are expressed in median (IQR). *MAP* Mean arterial pressure, *HR* Heart rate, *CO* Cardiac output, *SV* Stroke volume, *GALA* Global afterload angle, *TPU* Tissue perfusion unit, *PI* Perfusion index, *MFI* Microvascular flow index, *PPV* Percentage of perfused vessels, *StO2* Tissular saturation in O2, *THI* Tissular hemoglobin index, *TVD* Total vessel density, *PVD* Perfused vessel density, *HI* Heterogeneity Index. (*) = *p* < 0,05

**Fig. 2:**
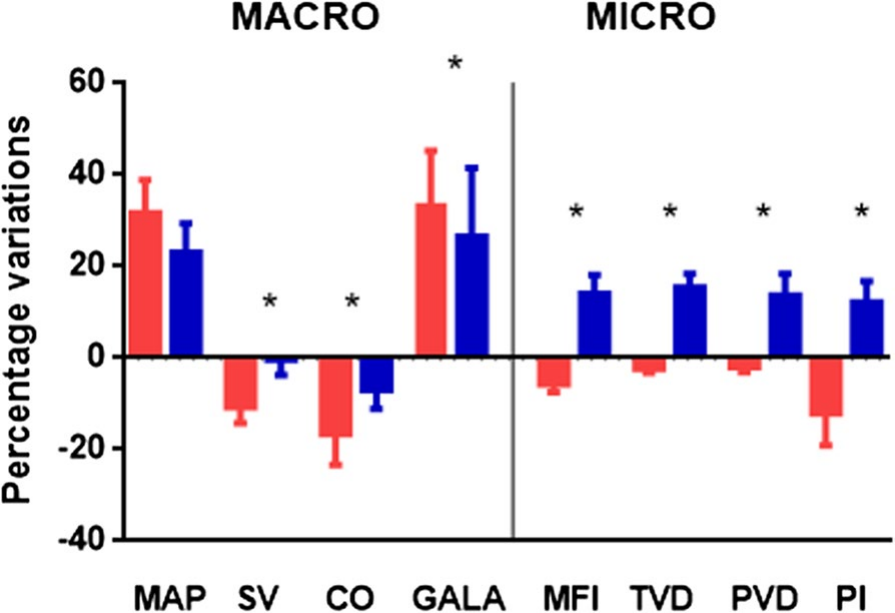
Comparison between the effect of a bolus (in red) and a continuous infusion (in blue). MAP = mean arterial pressure (mmHg); SV = stroke volume (mL); CO = cardiac output (L/min); GALA = global afterload angle (°); MFI = microvascular flow index; TVD = total vessel density (in mm2/mm2); PVD = perfused vessel density (mm2/mm2); PI = perfusion index

*Comparison between bolus and continuous infusion at peak of MAP:* For a smaller (but nonsignificantly different) increase in MAP (*p*=0,07), continuous infusion was associated with a more limited decrease in CO of 0,6 L/min (*p*< 0,001). As changes in HR were similar, this implies that differences in CO were attributed to differences in the decrease in SV. Indeed, we observed a smaller decrease in SV of 4 ml (*p*=0,009). This was associated with a smaller increase in the GALA of 7° (*p*=0,04). The results are exposed in Table 4 and Fig. 2.

**Table 4:** Comparison between bolus mode and continuous infusion.

	BOLUS	CONTINUOUS INFUSION	p
**MACROCIRCULATION**			
** MAP (mmHg)**	+21 (15 ; 30)	+ 16 (9 ;26)	0,07
** HR (/min)**	- 8 (-12 ; -6)	- 7 (-10 ; -4)	0,2
** CO (L/min)**	- 1,5 L (-1,1 ; -2,4)	- 0,9 ( -1,5 ; -0,2)	< 0,001
** SV (mL)**	-11 (-8 ; -20)	- 7 (-13 ; -3)	0,009
** GALA (°)**	+ 12 (6-15)	+ 7 (3-12)	0,04
**MICROCIRCULATION**			
** PHOTOPLETHYSMOGRAPHY**			
** PI (finger)(%)**	-12 (-24 ; 0)	+ 12 (4-20)	0,008
**VIDEOCAPILLAROSCOPY**			
** MFI**	-0,1 (-0,24 ; -0,06)	+0,3 (0,1-0,4)	0,03
** TVD (mm2/mm2)**	-0,2 (-0,2-0,7)	+ 2,3 (1,5-3,2)	0,002
** PVD (mm2/mm2)**	-0,36 (-1,5 ; -0,8)	+1,44 (-0,7 ; +3)	0,01
** HI (%)**	-0.24 (-0,2 ; 0,36)	+0.04 (-0,49 ; 0,25)	0,08

*MAP* Mean arterial pressure, *HR* Heart rate, *CO* Cardiac output, *SV* Stroke volume, *GALA* Global afterload angle, *TPU* Tissue perfusion unit, *PI* Perfusion index, *MFI* Microvascular flow index, *PPV* percentage of perfused vessels, *TVD* Total vessel density, *PVD* Perfused vessel density, *HI* heterogeneity index

### Microcirculatory parameters

Intra- and interindividual variability for the assessment of the MFI score was good, with kappa coefficients of concordance of 0.82 and 0.87, respectively (*p*<0.05). Microcirculatory parameters before bolus and continuous infusion were not different.

*Effect of a bolus of norepinephrine *(Table 2)*:* Norepinephrine boluses led to a downward but not significant trend in MFI from 2,16 [1,72-2,64] to 2,06 [4] (*p* = 0,09) and TVD from 14,1 [1, 9-11, 11-16] to 13,9 [4, 7-11, 11-15] (*p*=0.22). PVD also increased significantly from 10,1 [5, 9-11] to 11,8 [1, 7-10, 10-13] mm2/mm2 (*p*=0,01). This was associated with a significant decrease in PI of -12 [1-9, 9-24] % (*p*=0,006). Concomitantly, we observed an early and transient increase in TPU at T30s (+14 [2, 2-9, 9-26] % (*p*=0,003)). No significant variation concerning the remaining parameters was observed.

*Effect of a continuous infusion *(Table 3)*:* Norepinephrine continuous infusions were associated with an upwards but nonsignificant trend in MFI from 1,8 [1,7-2] to 2,1 [4] (*p* = 0,07) and a significant increase in TVD from 15,1 [1-5, 5-14, 14, 15]  to 17,4 [6, 7, 7-15, 15-18] mm2/mm2 (p = 0,007). Concomitantly, PI also increased but not significantly by + 12 (4-20)%. In contrast, we observed a decrease in StO2 at thenar eminence (*p*=0,03).

*Comparison between bolus and continuous infusion at peak of MAP:* bolus and continuous infusion showed overall opposite variations in microcirculatory parameters, as MFI (-0,1(-0,24;-0,06) vs. +0,3(0,1-0,4), p=0,03), PI (-12(-24;0) vs. +12(4-20)%, *p*=0,008), TVD (-0,2(-0,2-0,7) vs. +2,3(1,5-3,2)mm^2^/mm^2^, *p*=0,002), for bolus and continuous infusion, respectively. The results are exposed in Table 4 and Fig. 2.

## Discussion

Our results showed that bolus and continuous infusion of norepinephrine had different macro- and microcirculatory effects.

1. At the macrocirculatory level, continuous infusion resulted in a smaller decrease in CO and SV and a smaller increase in afterload than bolus administration.
2. At the microcirculatory level, continuous infusion enhanced blood flow and vessel density, while these variables were impaired with a bolus.

These results on macro and microcirculation enlighten the potential benefits of a continuous infusion of norepinephrine rather than a bolus to treat anaesthesia-induced hypotension.

We believe these results are important : in daily practice, the use of vasopressors to correct IOH is still mainly administered in a bolus [13, 28, 29], as IOH often occur at specific and usually short operative times (postinduction period, prone positioning, transient increase in hypovolemia, etc.), where the result of a bolus of vasopressors provides a concrete response on ABP in terms of both effectiveness and practicality. In a recent study, among 410 anesthesiologists and anesthetic nurses, 90% of the responders used norepinephrine at low dilution (16 gammas/ml or less). 26% reported using exclusively boluses and 54% revealed using both bolus and continuous infusion [30].

Even though the level of MAP reached with a continuous infusion could be slightly lower, those results argue for the preferential use of a continuous infusion of norepinephrine, rather than a bolus, to treat IOH.

### Norepinephrine and macrocirculation

Our work shows that norepinephrine could have deleterious effects on macrocirculation. Its use leads to a substantial decrease in CO and SV and to increased arterial stiffness. This is in line with previously published studies [6, 7, 9]. However, it must be mentioned that those effects are described in a very specific population: patients undergoing a scheduled neurosurgery/neuroradiology procedure, without hypovolemia and any previous circulatory failure.

Interestingly, we found that continuous infusion might lead to a smaller decrease in CO and SV than bolus infusion. It would allow a lesser increase in arterial stiffness and a conservation of SV via a lesser increase in afterload reflected by GALA angle variations.

Mechanisms explaining norepinephrine’s effects on CO remain uncertain, but hypotheses could be made of an association between the following:

- Increased cardiac inotropism driven by Norepinephrine’s beta agonist effect
- Venous constriction, increased mean circulatory filling pressure and ventricular preload

These mechanisms must be essentially present in continuous infusion administration, and it can potentially be argued that the rapid and acute increase in MAP during bolus administration involves other compensatory and protective mechanisms, such as the baroreflex, which are not, or are less involved, in continuous infusion administration.

### Norepinephrine and microcirculation

Because of its α1-adrenoreceptor agonist properties, norepinephrine appears to lead to decreased tissue perfusion when a bolus is administered. This is consistent with the results published by Poterman et al. [13].

Interestingly, the effects of continuous perfusion did not seem to reproduce this deleterious effect, and instead we observed a tendency to improve microcirculatory flow and density. In a prospective study, Flick et al. were using norepineprhrine in continuous infusion on patients having surgery with moderate blood loss. The moderate blood loss and the introduction of catecholamines in continuous infusion had not detrimental effect on sublingual microcirculation either [31].

These results could be explained by various reasons:

- First, as already discussed, the rapid increase in MAP during the bolus administration might enhance an adaptive mechanism of protection and autoregulation, such as intense vasoconstriction.
- Second, the bolus mode of administration provokes a greater increase in norepinephrine plasma concentration levels compared to continuous administration, even if the total dose (10 vs. 16 gammas) is smaller. This could be at the origin of a greater stimulation of α1-adrenoreceptors and precapillary sphincters.
- Third, CO is a major determinant of tissue perfusion even if mechanisms of autoregulation exist at the cellular scale.

At the end of the process, the combined decrease in CO and greater tissue vasoconstriction may explain the decrease in flow and vessel density.

StO2 and videocapillaroscopy seem to show contradictory results when looking at continuous infusion effects.

One could argue that variations in regional blood flow at the administration of norepinephrine should be considered differently depending on the organ of focus. Consequently, we can logically suppose that norepinephrine leads to cutaneous vasoconstriction, which is responsible for the decrease in microcirculatory parameters reflecting capillary density in that particular territory (StO2 and/or THI). On the other hand, in the central location (as the sublingual area), the effects could be opposite, with a central redistribution of blood consecutive to peripheral vasoconstriction.

### Study limits

This study had some limitations

This is an observational study with a small sample size of patients, and clinical implications should be carefully considered. The smaller size of continuous infusion sequences analysed in videocapillaroscopy could be responsible for selection bias. This small sample is partially due to difficulties encountered during recording attempts, with restricted access to the mouth. These limitations are also linked to the device (which requires a very precise use and permanent removal of saliva) and explain why videocapillaroscopy, even if very intriguing, is not an easily used bedside tool for daily practice, at least in the operating room. Difficulty in recording is accountable to a certain power in our study.

Our results cannot be generalized to other types of shock as we studied a population of carefully selected patients who only presented anaesthesia-induced hypotension. As previously published, the effects on macro- and microcirculatory parameters of norepinephrine could be different depending on the haemodynamic state of the patient before infusion [32, 33].

The absence of randomization might be responsible of selection bias. Because the intervention (bolus or continuous infusion) is at the discretion of the anaesthesiologist, boluses and infusions could be compared in different physiologic situations.

As mentioned earlier, the increase in MAP appears to be greater, although not significant, with the bolus mode of administration, which could have influenced our results on the macro- and microcirculatory parameters. The differences observed may be slightly overestimated.

It could also be argued that the comparison between different moments (T60, T180 and T300 seconds) is not relevant. More than the comparison of two moments (T60 vs T300), we tried to compare two ways of administrating the same drug when the effect on MAP was at its maximal. Those times were decided before we started the protocol, and are based on a study we are about to publish, focusing precisely on pharmacodynamics/pharmacokinetics effects of norepinephrine.

We decided to put in light the results offered by the videocapillaroscopy more than the other monitors : PI seems to have the same variations but it is true that laser Doppler, tissular CO2 and the StO2 are not always going the same way. The main reason we decided to present the results that way is that today videocapillaroscopy is seen as the gold standard monitor of microcirculation since several years, and the most precise device, even if recording might be difficult. The recent publication of the consensus of 2018 is a reflection of the interest showed by the scientific community. We decided to present all the results, even those evolving in a opposite way, because we found it interesting for the readers. Our final experience remains that some of the monitors we used (tissular CO2, StO2) have not the capacity of detecting such rapid moves as we wanted to study. Their time of reaction doesn’t allow to make a sure and certain conclusion.

Last, we do not report the post-operatives outcomes of the patients we studied. The reason is the study design was not powered for this, as it was a preliminary work. Our results pave the way for an randomized trial.

## Conclusion

These results on macro and microcirculation enlighten the potential benefits of a continuous infusion of norepinephrine rather than a bolus to treat anaesthesia-induced hypotension.

Indeed, continuous infusion of norepinephrine appears to preserve microcirculatory flow and capillary density and results in a smaller decrease in CO compared with the bolus mode of administration. Future studies are still warranted to confirm these results in a larger sample of patients.

## Data Availability

Data will be available on reasonable request formulated to corresponding author.
